# Survival-associated N^6^-adenosine methyltransferase signatures in lung squamous cell carcinoma and clinical verification

**DOI:** 10.1186/s12885-021-08939-6

**Published:** 2021-11-24

**Authors:** Jialin Qu, Li Wang, Man Jiang, Zhimin Wei, Guangming Fu, Xiaochun Zhang

**Affiliations:** 1 Department of Radiation Oncology, Shandong Cancer Hospital and Institute, Shandong First Medical University and Shandong Academy of Medical Science, Jinan, 250117 China; 2 Department of Oncology, Jiujiang University Affiliated Hospital, Jiujiang, 332000 China; 3 Cancer Precision Medicine Center, The Affiliated Hospital of Qingdao University, Qingdao University, Qingdao, 266003 China; 4 Pathology Department, The Affiliated Hospital of Qingdao University, Qingdao University, Qingdao, 266003 China

**Keywords:** N^6^-methyladenine, Prognostic model, Lung squamous cell carcinoma, Bioinformatic analysis, Epigenetics

## Abstract

**Background:**

N^6^-methyladenine (m^6^A) is the most common modification of mRNA and IncRNA in higher organisms. m^6^A has been confirmed to be related to the formation and progression of tumors and m^6^A-related genes can be used as prognostic biomarkers in a variety of tumors. However, there have been no similar studies on lung squamous cell carcinoma. The main purpose of this study was aimed to explore the differential expression of m^6^A-related genes in lung squamous cell carcinoma tissues and its relationship with patient clinical prognosis.

**Methods:**

We integrated three m^6^A writers that catalyze the methylation of adenine on mRNA molecules. The training set including 501 patients with LUSC was collected from The Cancer Genome Atlas (TCGA) database and the test set including 181 patients with LUSC was collected from the Gene Expression Omnibus (GEO) database. Based on the expression level of the m^6^A methylase gene, we established a tumor subgroup and risk-prognosis model to quantify the risk index and long-term patient prognosis, which were confirmed by principal component analysis (PCA) and receiver operating characteristic (ROC) curve analysis. After lung squamous cell carcinoma tissue specimens were obtained during surgery, immunohistochemistry (IHC) was used to verify the results in vitro.

**Results:**

The results of the study showed that the expression of the three m^6^A methylases in tumor tissues and normal tissues was significantly different (*P* < 0.05). The survival-prognostic model based on METTL3 gene expression showed better predictive performance (AUC: 0.706). Patients in the high-risk and low-risk groups exhibited significant differences in terms of survival time and 5-year and 10-year survival rates. Immunohistochemistry revealed that patients with high METTL3 expression exhibited a longer survival time than those with low METTL3 expression.

**Conclusions:**

Our study showed that the molecular phenotype based on the expression of METTL3 may be an independent risk factor affecting the prognosis of lung squamous cell carcinoma. These findings not only prove the important role of m^6^A methylase in lung squamous cell carcinoma, but are also expected to provide more accurate prognostic assessment and individualized treatment for patients with lung squamous cell carcinoma.

## Introduction

Lung cancer is the malignant tumor with the highest incidence and fatality rate in the world. In 2020, lung cancer is expected to account for approximately 23% of all cancer-related deaths [1]. Eighty-five percent of lung cancers are nonsmall cell lung cancers (NSCLCs) according to histological classification, including lung adenocarcinoma (LUAD) and lung squamous cell carcinoma (LUSC) [2]. Currently, a variety of driver gene mutations such as EGFR, BRAF, ERBB2 or rearrangement of ALK or ROS1 are found in lung adenocarcinoma and the corresponding small molecule tyrosine kinase inhibitors (TKIs) have been put into clinical application, which prolong the median survival of patients with advanced lung adenocarcinoma [3]. However, lung squamous cell carcinoma, which accounts for 40 to 51% of nonsmall cell lung cancers, lacks driver gene mutations that can be used as drug targets. Except for a small number of early-stage patients who can be surgically removed, most advanced patients require platinum-based chemotherapy [4]. In the context of the continuous updating of tumor treatment and improvement of survival rates, lung squamous cell carcinoma has not made breakthrough progress in decades except immunotherapy and its 5-year survival rate is only 5% [5]. In recent years, incresing evidence has shown that the occurrence and development of lung squamous cell carcinoma is not only dependent on genetic variation, but also affected by epigenetic abnormalities [6].

Epigenetics can regulate gene expression at multiple levels, including not only DNA methylation, histone acetylation and chromatin remodeling, but also post-transcriptional modification of RNA [7]. m^6^A modification is the most common internal modification of eukaryotic messenger RNA (mRNA), which is essential for cell survival and development [8]. The methylation of m^6^A is catalyzed by methyltransferase complexes including methyltransferase-like 3 (METTL3) and methyltransferase-like 14 (METTL14) and its cofactor Wilms tumor I associated protein (WTAP). It is a reversible and dynamic RNA modification that can affect thousands of mRNAs and non-coding RNAs in cells [9]. In recent years, a lot of studies have found that m^6^A methyltransferase plays an important role in the occurrence and prognosis of malignant tumors. Overexpression of METTL14 can promote the proliferation and metastasis of liver cancer cells, leading to a worse prognosis for patients [10]. Studies have found that the METTL3 expression is upregulated in human lung adenocarcinoma cell lines [11]. Researchers have found that METTL3 is associated with longer survival in patients with lung adenocarcinoma, and METTL3 can be used as a biomarker to predict the prognosis of lung adenocarcinoma [12]. Another study found that upregulation of METTL3 is not only associated with longer overall survival (OS) in lung adenocarcinoma, but also associated with better recurrence-free survival (RFS) [13]. This suggests that m^6^A methyltransferase may be a new biomarker used to effectively predict the prognosis of tumors. However, as the second most pathological subtype of lung cancer, lung squamous cell carcinoma, such research is still relatively rare.

Traditional tumor staging aimed to judge the prognosis and whether patients can receive surgical treatment usually takes tumor size, lymph node and distant metastasis as key indicators [14]. However, due to individual differences and the heterogeneity of tumor cells, patients with the same tumor stage often have completely different prognoses [15]. Recent studies have indicated that different gene or molecular expression levels are also critical factors affecting the prognosis of lung cancer. This implies that the level of gene or molecular expression is of vital significance in assessing lung cancer prognosis. In this article, we systematically analyzed the differential expression of m^6^A methyltransferase in normal tissues and lung squamous cell carcinoma tissues as well as the correlation with clinical features. Furthermore, we distinguished different tumor subgroups and constructed a risk-prognosis model based on differences in gene expression, which were verified in other databases and clinical specimens to show the predictive value of m^6^A methyltransferase for lung squamous cell carcinoma prognosis.

## Methods

### Data download and integration

We downloaded an RNA-seq transcriptome profiling dataset including 49 normal tissues and 502 LUSC tissues and matching clinical information including age, sex, stage, tumor-node-metastasis classification over survival time and survival status from TCGA (https://portal.gdc.cancer.gov/). After deleting some invalid data with incomplete information, a total of 478 LUSC patients’ transcriptome and clinical data from the TCGA database were included in the training set for subsequent analysis (Table 1).

**Table 1:** **Clinical information of patients with LUSC from the TCGA database**

Clinical information	Number	Percentage
**Total cases**	478	100
**Survival status**		
Alive	290	60.67
Dead	188	39.33
**Over survival time**		
< 1 year	142	29.71
1–5 years	273	57.11
> 5 years	63	13.18
**Age**		
< 65	165	34.52
≥ 65	313	65.48
**Gender**		
Male	352	73.64
Female	126	26.36
**Stage**		
StageI	233	48.74
StageII	157	32.85
StageIII	82	17.15
StageIV	6	1.26
**Tumor**		
T1	108	22.59
T2	281	58.79
T3	67	14.02
T4	22	4.60
**Node**		
N0	308	64.44
N1	127	26.57
N2	38	7.95
N3	5	1.04
**Metastasis**		
M0	397	83.05
M1	81	16.95

To further verify the accuracy of the results, we also selected data from other databases for analysis. mRNA expression of seven NSCLC test set (GSE12472, GSE8894, GSE4882, GSE41271, GSE30219, GSE42127, GSE37745) was obtained from the GEO database (https://www.ncbi.nlm.nih.gov/geo/). We selected 181 eligible patients with LUSC from these data sets. (Table 2).

**Table 2:** Clinical information of patients with LUSC from the GEO database

Clinical information	Number	Percentage
**Total cases**	181	100
**Survival status**		
Alive	74	40.88
Dead	107	59.12
**Over survival time**		
< 1 year	38	20.99
1–5 years	97	53.59
> 5 years	46	25.42
**Age**		
< 65	78	43.09
≥ 65	103	56.91
**Gender**		
Male	138	76.24
Female	43	23.76

### Bioinformatic data analysis

After gene name conversion and correction of transcriptome data, we extracted m^6^A methyltransferase expression data of LUSC patients from the TCGA and GEO databases. Based on the classification of tumor tissues and normal tissues in TCGA sets, we analyzed the expression of m^6^A methyltransferase. Heatmaps were drawn to visualize the differential expression of m^6^A methyltransferase between tumor and normal tissues.

To explore the role of three different proteins that make up the m^6^A methyltransferase complex in lung squamous cell carcinoma, the “Consensus Cluster Plus” package was selected to remove similar samples and classify tumor samples into different molecular subtypes according to the expression of three genes. We used principal component analysis (PCA) to verify the clustering effects and rationality. By combining clinical information, survival curves and clinically correlated heatmaps were created to analyze survival differences between subgroups and the relationship between subgroups and clinical features.

To screen m6A methyltransferase genes highly related to prognosis and assess the prognostic evaluation, univariate Cox regression analysis was performed.

Risk score = $$ {\Sigma}_{\mathrm{i}=1}^{\mathrm{n}}\ {\mathrm{Coefi}}^{\ast}\mathrm{Xi}. $$

(Coefi is the coefficient of each selected gene, Xi is the expression value of METTL3.)

From this formula, each patient can get a specific risk score. According to the median value, we defined patients whose risk score ≥ median value into high-risk group, and patients whose risk score < median value into low-risk group (Table 3). Then Kaplan-Meier analysis was performed to display the prognostic performance of risk score model in both training and test cohorts. A receiver operating characteristic (ROC) curve was collected to verify the effectiveness of the model. Furthermore, we used multivariate Cox regression to investigate the independent prognostic role of the risk model.

**Table 3:** Overall survival time and clinicopathologic factors between high-risk group and low-risk group in TCGA database

Clinical information	High-risk group	Low-risk group
**Total cases**	239(50%)	239(50%)
**Survival status**		
Alive	131(55%)	159(67%)
Dead	108(45%)	80(33%)
**Overall survival time**		
< 1 year	73(31%)	42(17.5%)
1–5 years	134(56%)	152(63.5%)
> 5 years	32(13%)	45(19%)
**Age**		
< 65	80(33%)	93(36%)
≥ 65	159(67%)	166(64%)
**Gender**		
Male	68(28%)	55(23%)
Female	171(72%)	184(77%)
**Stage**		
StageI	121(51%)	113(47%)
StageII	72(30%)	79(33%)
StageIII	40(17%)	43(18%)
StageIV	6(2%)	4(2%)
**Tumor**		
T1	63(26%)	51(21%)
T2	131(55%)	145(61%)
T3	31(13%)	35(15%)
T4	14(6%)	8(3%)
**Node**		
N0	152(64%)	151(63%)
N1	63(26%)	63(26%)
N2	20(8%)	19(8%)
N3	4(2%)	6(3%)
**Metastasis**		
M0	197(82%)	192(80%)
M1	42(18%)	47(20%)

In the analysis results of the TCGA database, we found that METTL3 expression was the most significantly different gene between the two tumor subtype groups and that the survival time of patients in the METTL3 high expression group was notably longer than those in the METTL3 low expression group. Therefore, we only analyzed the differential expression of a single gene, METTL3, in the GEO database to verify the results we found in the TCGA database.

Last, in addition to the differential expression of m^6^A methyltransferase, we attempted to clarify the possible molecular mechanisms of the overall survival-associated m^6^A methyltransferase. Through Gene Ontology (GO) and Kyoto Encyclopedia of Genes and Genomes (KEGG) analysis, we obtained the biological functions and signaling pathways of METTL3, which can provide a preliminary explanation for our results.

### Immunohistochemistry

70 surgical specimens of LUSC from the Affiliated Hospital of Qingdao University and performed immunohistochemical staining. Immunohistochemistry (IHC) analysis to detect the expression level of METTL3 was performed using GTVision™ III Detection Systems (Genetech, Shanghai, China) and METTL3 antibody (ab195352) according to the manufacturer’s instructions. Two pathologists who were unaware of the patient’s clinical information evaluated the immunohistochemical sections. When their opinions disagree, the third pathologist conducts an independent evaluation. They observed ten optical fields under a high-power lens (× 400), and calculated the degree of staining of positive cells and the percentage of positive cells. The final staining score was judged by the following criteria: staining intensity score. 0 (without coloration, no staining), 1 (pale yellow, weak), 2(brownish-yellow, moderate) or 3 (brown color, strong); staining area score, 0 (≤10% positive staining), 1 (11–25% positive staining), 2 (26–50% positive staining), 3 (51–75% positive staining) and 4 (≥75% positive staining). The final score is the sum of the staining intensity and staining area [16].

### Software for statistical analysis

RNA-seq transcriptome data from TCGA and GEO were processed by R v 4.0.2 (www.r-project.org/). We applied the Wilcoxon test to compare the expression differences of m^6^A methyltransferase genes between tumor and normal tissues. The duration of the patient from the date of diagnosis to the date of death was defined as the overall survival time. The overall survival time of the two tumor subgroups was evaluated by the Kaplan-Meier method and visualized by the survival curve (SPSS 23.0 software). The correlation between tumor subtypes or risk groups and clinical characteristics was judged by the chi-square test. A *P* value < 0.05 indicated statistical significance.

## Results

### The m^6^A methyltransferase gene shows a significant expression difference between lung squamous cell carcinoma and normal tissues

**Fig. 1:**
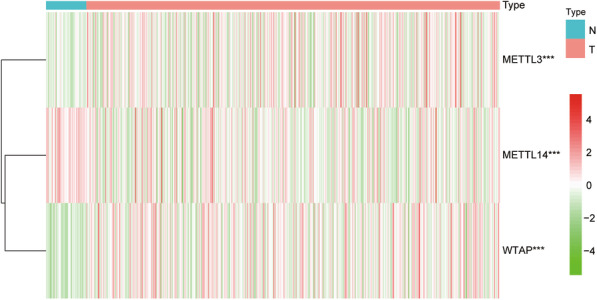
Differential expression of the three proteins composing the m^6^A methyltransferase complex in LUSC and normal tissues. (*, P < 0.05; **, P < 0.01; ***, P < 0.001)

According to the RNA-seq transcriptome data from the TCGA database, we analyzed the expression levels of m^6^A methyltransferase genes from tumor and normal tissues. The analysis results showed that the expression of METTL3 and WTAP in LUSC tissues was significantly higher than that in normal tissues, while METTL14 expression was low in tumor tissues (*P* < 0.05) (Fig. 1), which suggests that m^6^A methylation modification plays an indispensable role in the occurrence and development of LUSC.

### Two tumor subtypes based on the expression level of m^6^A methyltransferase have different prognoses

**Fig. 2:**
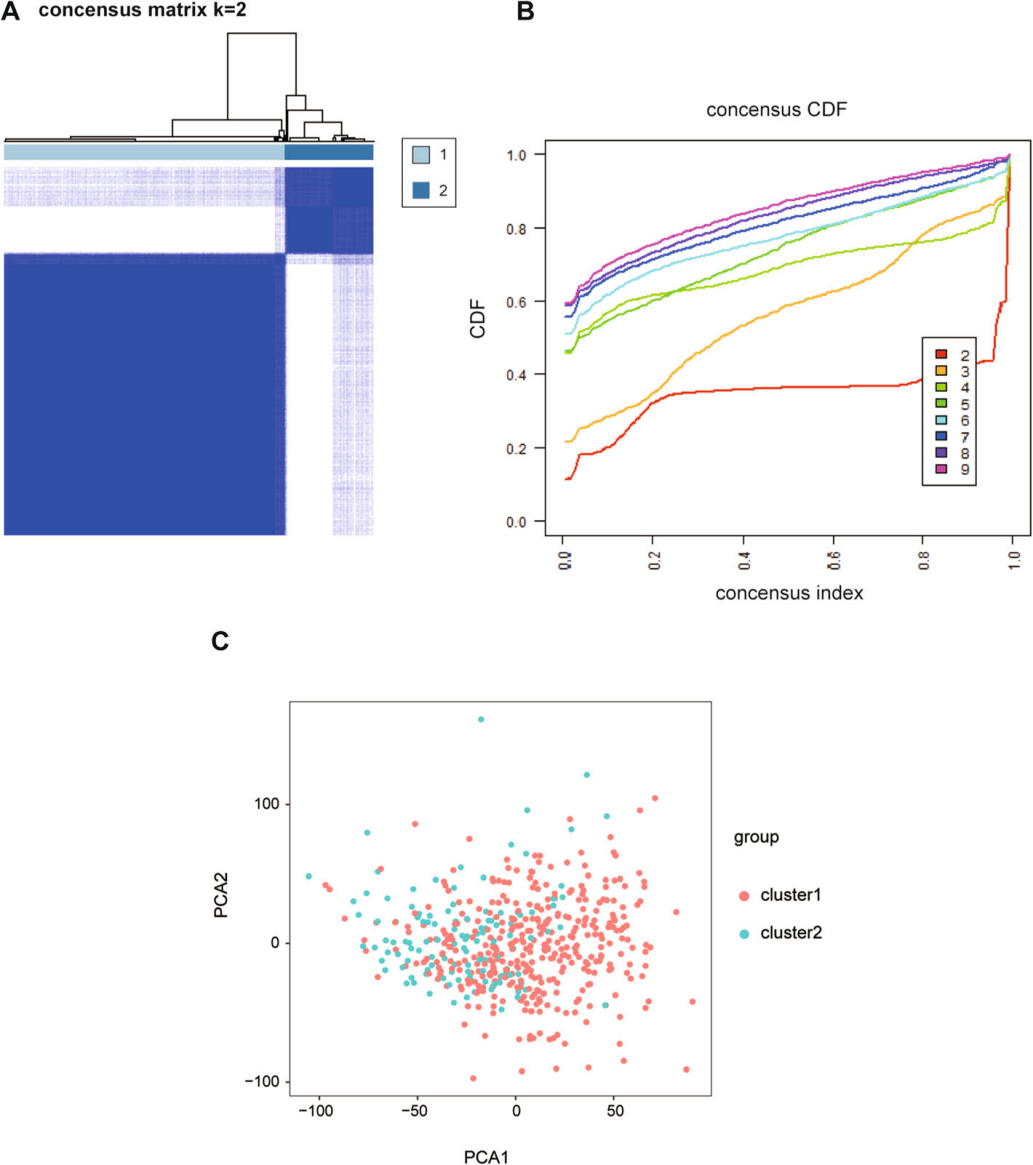
PCA and cluster model were used to divide all lung squamous cell carcinoma patients into two molecular subtypes based on the m^6^A methyltransferase gene expression. (A, B, C) PCA: principal component analysis

After deleting samples from normal tissues, the “Consensus Cluster Plus” package was used to classify 478 LUSC samples into different subtypes based on the expression of three m^6^A methyltransferases. Taking into account the clustering stability and the number of samples between each group, we believe that it is appropriate to divide all LUSC samples into two subgroups. In the clustering model of LUSC, we observed partial overlap between the two subgroups according to principal component analysis (PCA), but the overall dispersion was acceptable (Fig. 2A-C). This evidence implies that our classification method based on m^6^A methyltransferase is appropriate.

**Fig. 3:**
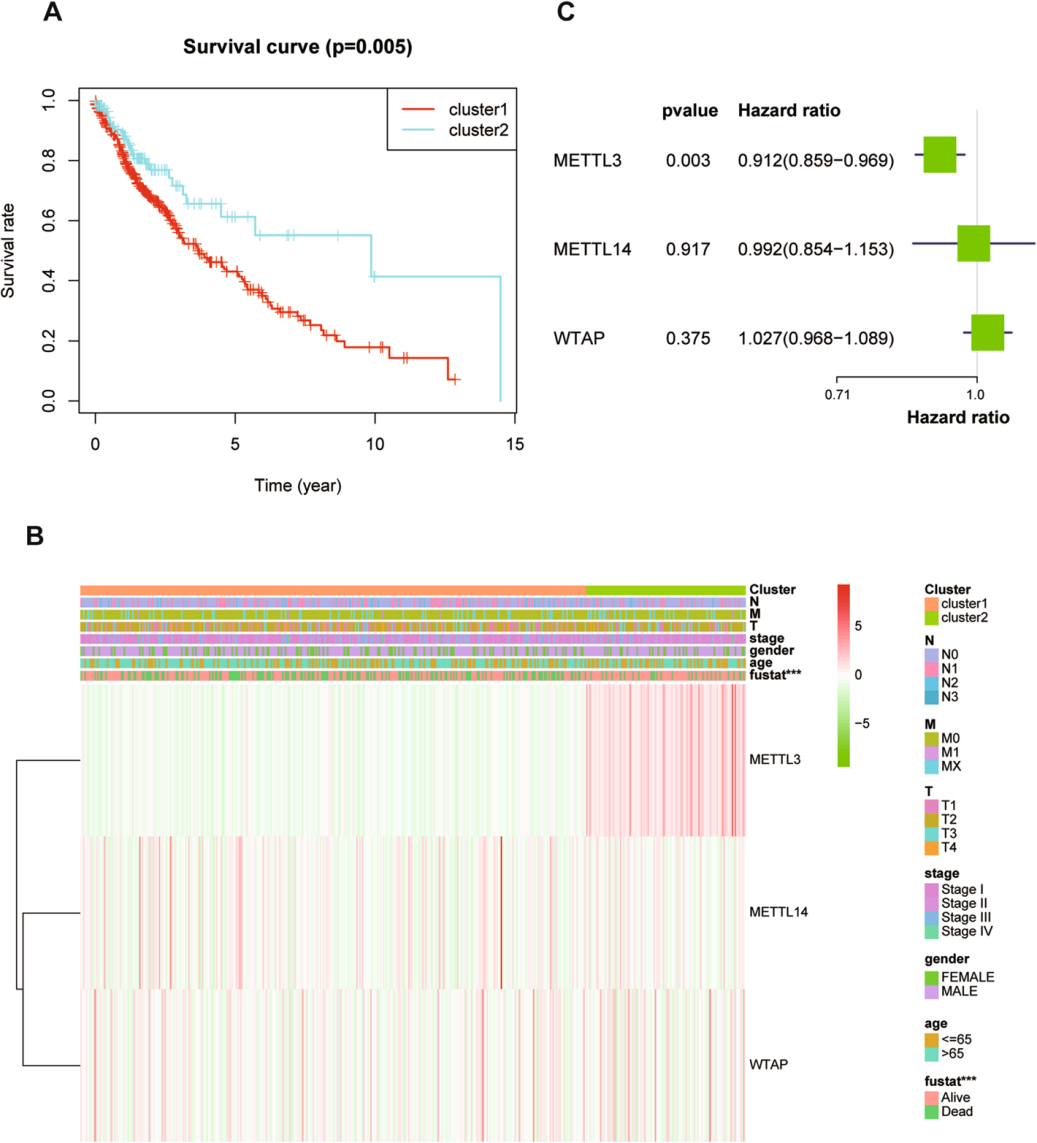
(A) Survival curve and clinical characteristics of the tumor subtypes based on the m^6^A methyltransferase from TCGA database. (B) Clinical characteristics of the tumor subtypes based on the m^6^A methyltransferase from TCGA database. (C) Univariate Cox regression predicts the relationship between gene expression and prognosis

To more intuitively reflect the relationship between tumor subtypes and prognosis or clinical characteristics, we conducted survival analysis and drew a clinical correlation heatmap. The survival curve showed that the overall survival time of the two groups was significantly different (*P* = 0.005) (Fig. 3A). The clinical correlation heatmap further points to the relationship between the gene expression of m^6^A methyltransferase and clinical characteristics. Among the three genes of the m^6^A methyltransferase complex, METTL3 is highly expressed in cluster 2 and positively correlated with overall survival time. (*P* < 0.01) (Fig. 3B).

### Establishment of a risk-prognosis model to predict the risk factors and prognosis of LUSC

**Fig. 4:**
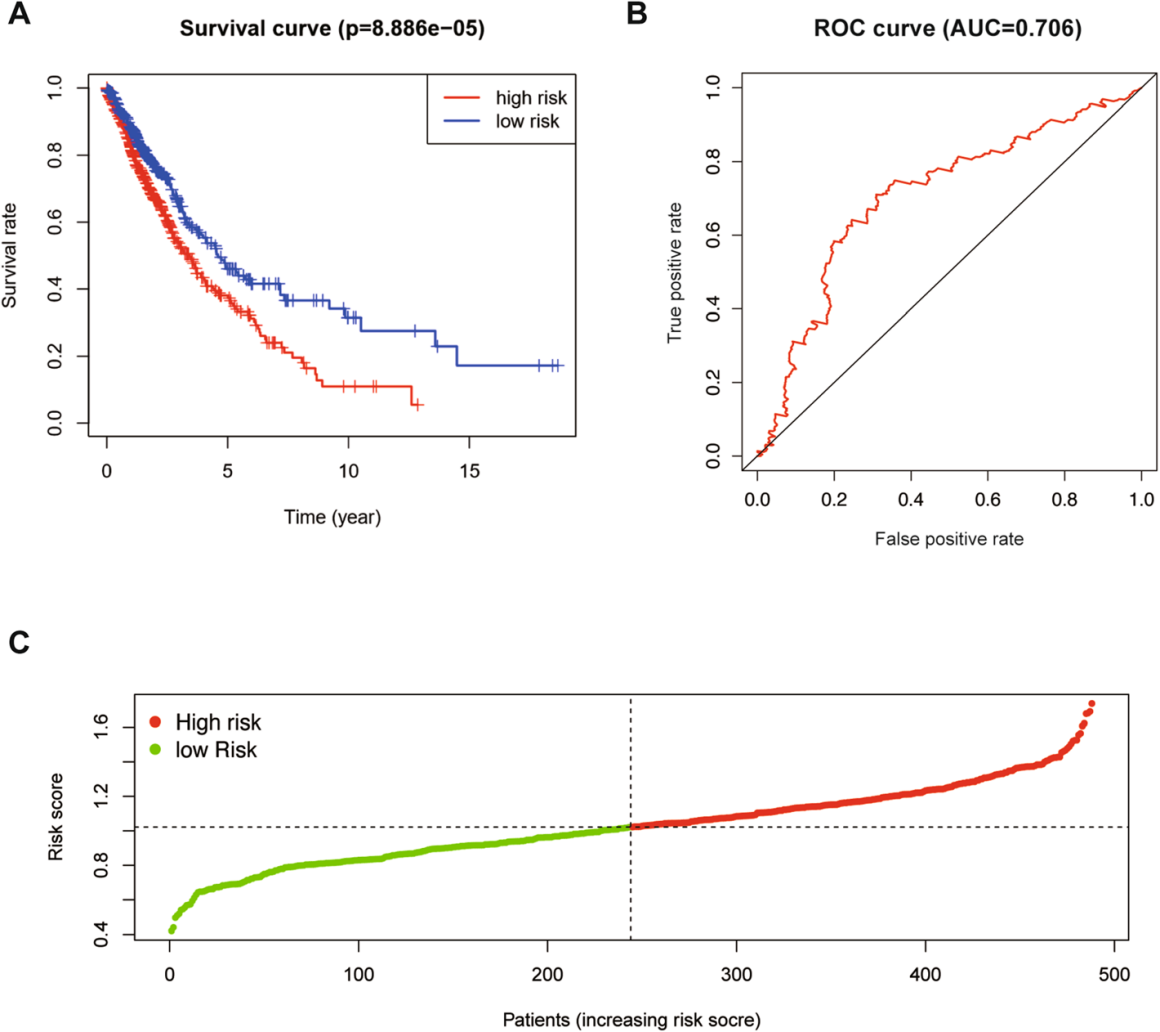
(A) The training set based on TCGA database. K-M plot of the prognostic model constructed with METTL3 for LUSC patients. (B) ROC curves with calculated AUCs of the prognostic model. (C) Risk score distribution of LUSC with different risks

To better understand the role of m^6^A methyltransferase in evaluating the prognosis of LUSC, we created a Cox model based on the expression of m^6^A methyltransferase and overall survival time in the TCGA database. Univariate Cox regression analysis was used to screen genes closely related to prognosis. The analysis results showed that METTL3 is closely related to the prognosis of patients and that its expression is negatively correlated with prognosis, which shows that METTL3 is a low-risk gene in LUSC and can be used as a basis for patient risk scores (Fig. 3C) (HR:0.912, *P* = 0.003). Based on the risk score, we divided all patients into two subgroups: a high-risk group and a low-risk group. Finally, a survival-prognosis model was established, and Kaplan-Meier survival analysis of the model showed that the survival period of patients in the low-risk group was significantly longer than that of the high-risk group, which implied a remarkable ability in differentiating good or poor clinical outcomes between the two subgroups (Fig. 4A, C, D) (*P* < 0.01). To evaluate the effectiveness of the model, we also generated the ROC curve and calculated the areas under the curve (AUCs). As shown in the figure, the AUC of the model exceeded 0.7, showing a strong predictive ability for the prognosis of patients with LUSC (Fig. 4B).

**Fig. 5:**
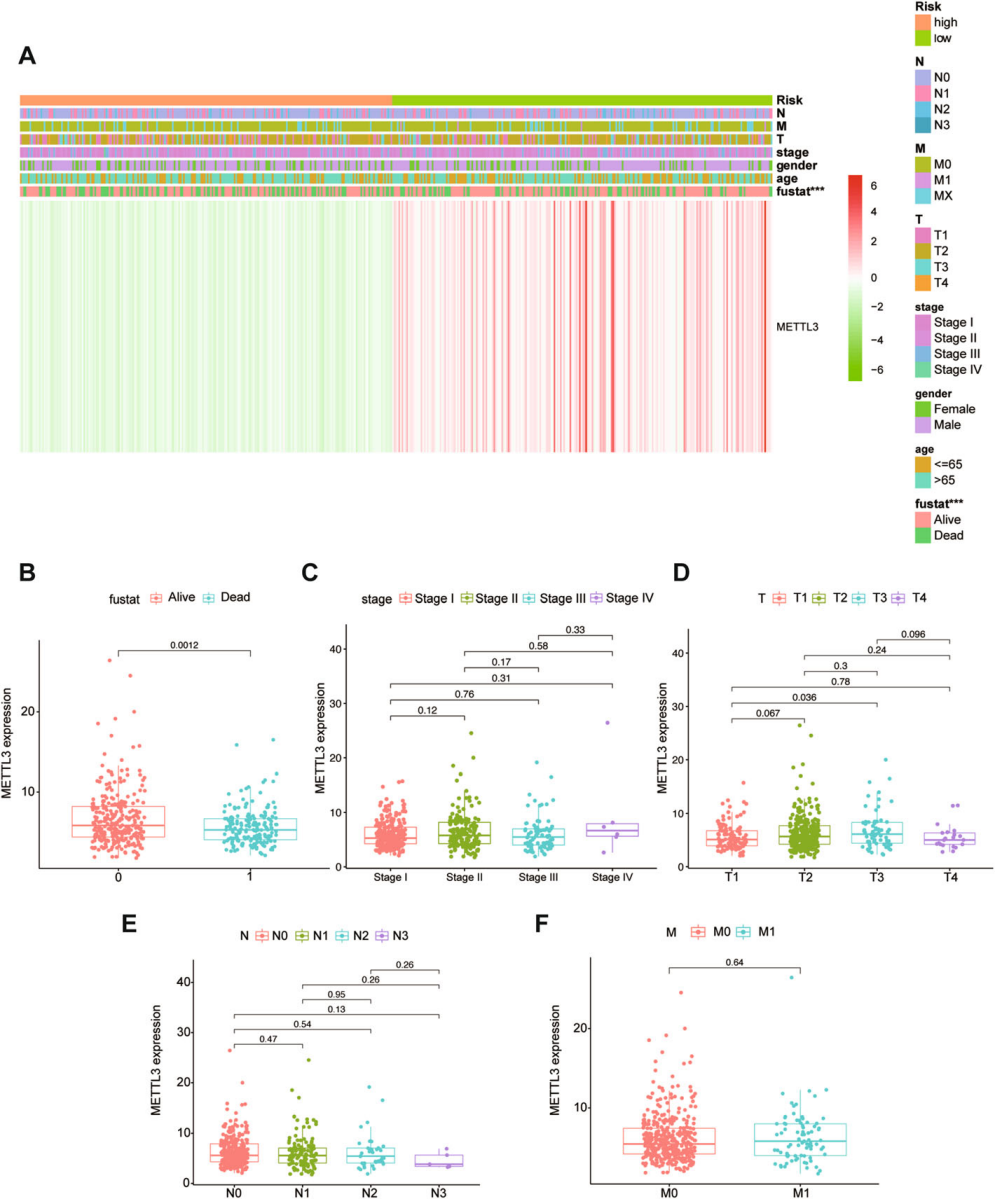
(A) The relationship between the risk group constructed based on the expression of METTL3. (B-F) Clinical characteristics of LUSC in the TCGA database. (*, *P* < 0.05; **, *P* < 0.01; ***, *P* < 0.001)

In addition, we also produced heatmaps and scatter plots to explore whether there is a relationship between METTL3 expression and the clinical characteristics of patients with LUSC. The results show that the expression of METTL3 is significantly related to the prognosis of LUSC (Fig. 5A, B) (*P* < 0.01), but not to clinical indicators such as stage, tumor size, lymph node and distant metastasis (Fig. 5C-F)

**Fig. 6:**
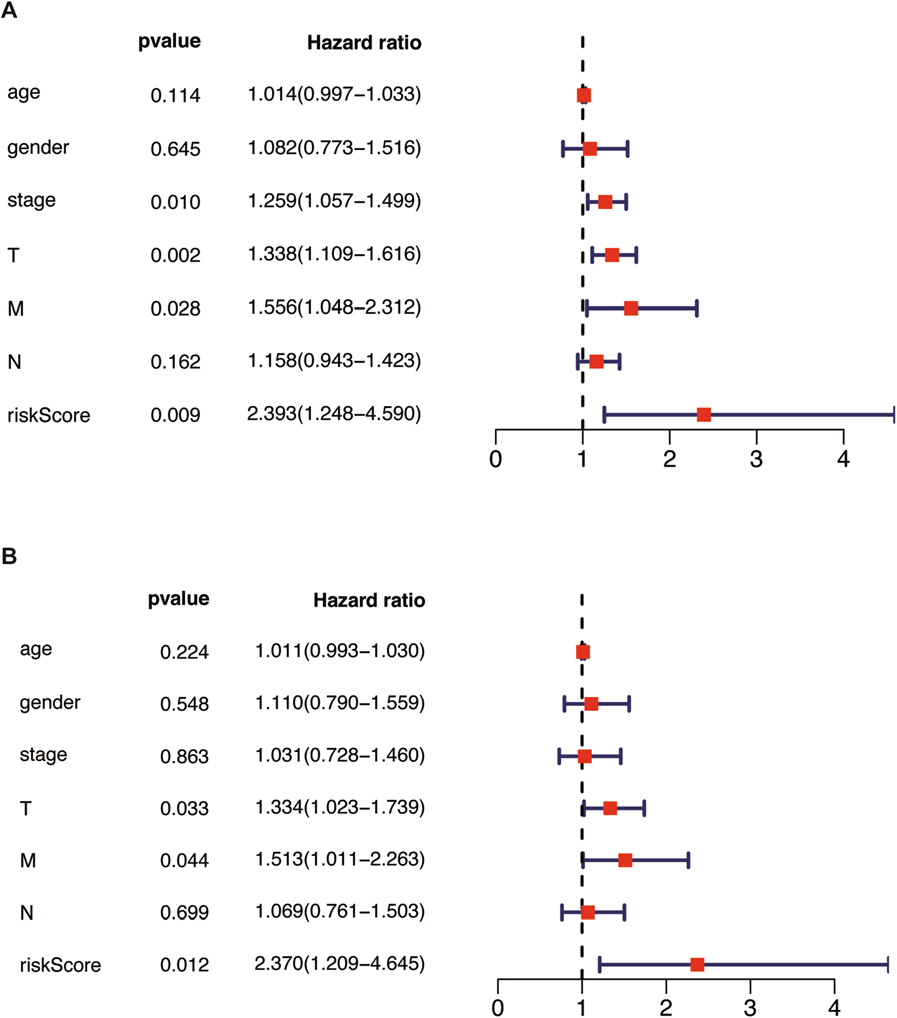
(A) Independent prognostic analysis of training set. Univariate independent prognostic analysis. (B) Multivariate factor independent prognostic analysis

We further used univariate and multivariate independent prognostic analyses to evaluate the predictive value of the prognostic model. All independent prognostic analysis results were consistent, which shows that the METTL3 gene can be independent of other clinical characteristics as an independent prognostic factor. High-risk patients with low METTL3 expression may have a short survival period, suggesting a poor prognosis (Fig. 6).

### The reliability of the risk-prognosis model was verified by the GEO database

**Fig. 7:**
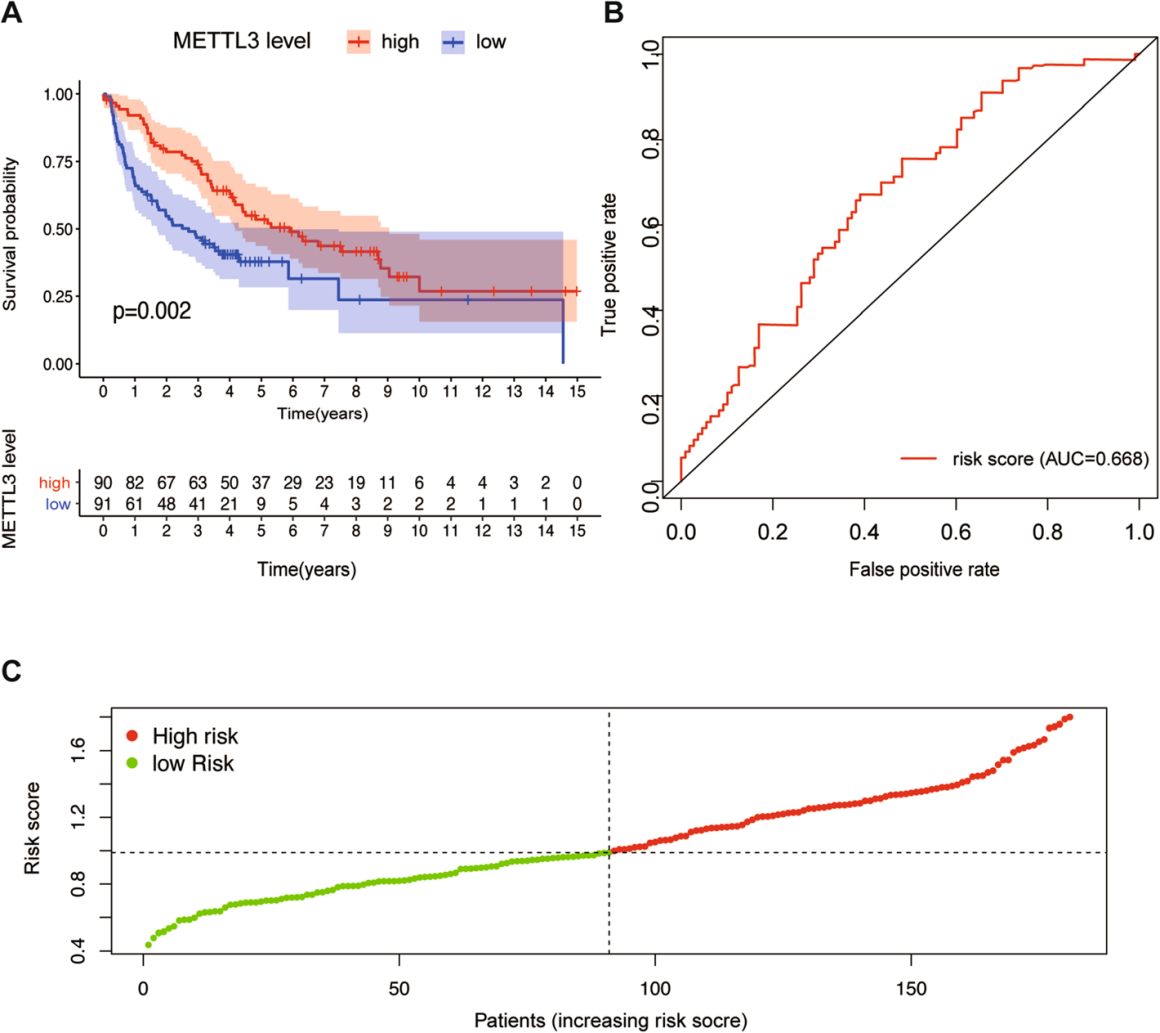
(A) The test set based on GEO database. The K-M plot of prognostic model constructed with METTL3 for LUSC patients. (B) ROC curves with calculated AUCs of prognostic model. (C) Risk score distribution of LUSC with different risks

To verify the credibility of the risk-prognosis model constructed by the TCGA database, we repeated modeling using data from other databases. We selected seven NSCLC datasets that can provide basic clinical information including overall survival time and survival status from the GEO database and extracted gene expression data and clinical information of 181 patients with LUSC. By counting the expression of METTL3, all patients were divided into high-risk and low-risk groups and survival analysis was performed. The results of survival analysis showed that the overall survival time of LUSC patients with high METTL3 expression was significantly prolonged (Fig. 7A, C, D*)* (*P* = 0.002), which confirmed the accuracy of the prognostic model based on METTL3. However, the predictive effect of this prognostic model was slightly lower than that of the model based on the TCGA database (AUC = 0.668) (Fig. 7B).

**Fig. 8:**
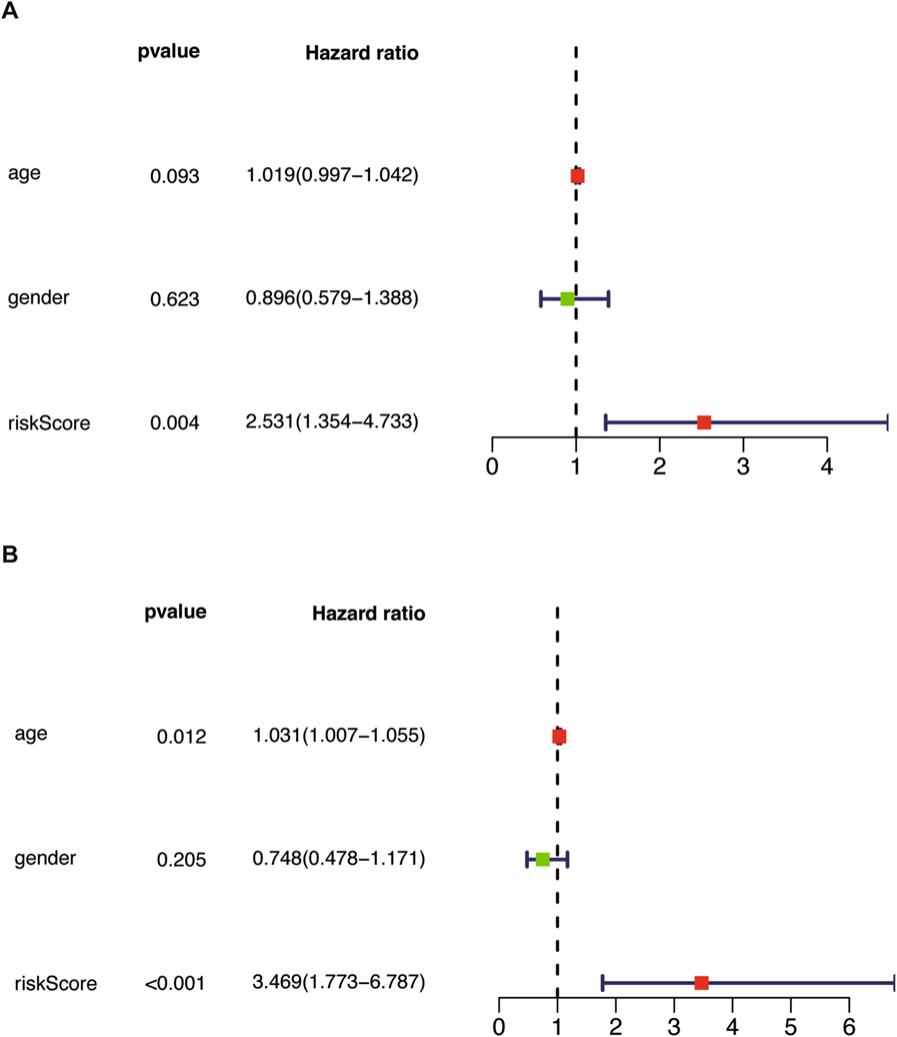
Independent prognostic analysis of test set. (A) Univariate independent prognostic analysis. (B) Multivariate factor independent prognostic analysis

The clinical information obtained in the GEO database is limited, including only the age, sex and overall survival time of the patients. We still performed univariate and multivariate independent prognostic analyses to verify the accuracy of the model. The results of independent prognostic analysis were basically consistent with the results of the TCGA database, which supports the risk-prognosis model based on the expression of METTL3 (Fig. 8). In general, the results obtained on the training set were successfully verified in the test set, which suggests that METTL3 is an effective prognostic predictor of LUSC.

### Functional enrichment analysis of METTL3

**Fig. 9:**
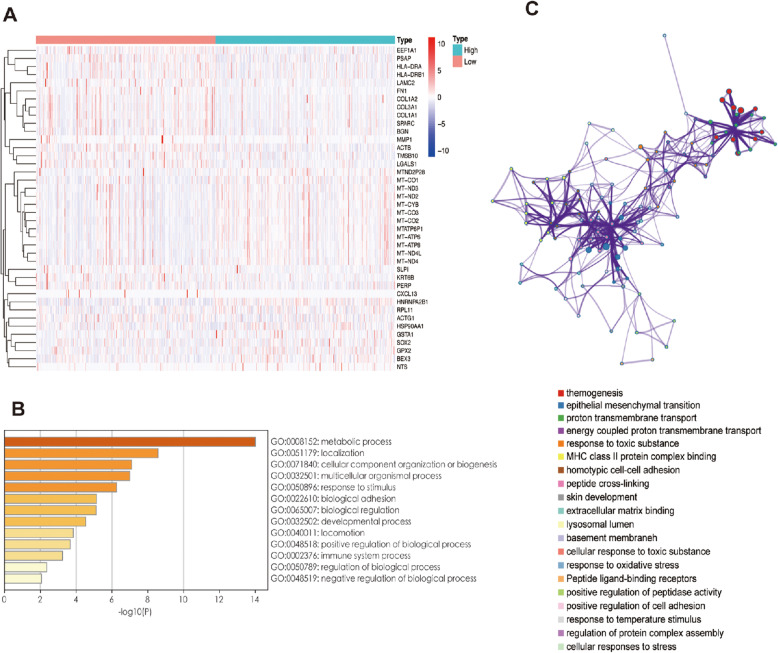
Function analysis of METTL3. (A) Heatmap of differential genes in METTL3 high and low expression groups. (B) Gene ontology. (C) Kyoto Encyclopedia of Genes and Genomes

After understanding that METTL3 is related to the prognosis of LUSC and creating a risk model, we attempted to further clarify how METTL3 affects the patient prognosis. We first found the 40 genes most closely regulated by METTL3 in the tumor samples of the TCGA database and displayed them in the form of a heatmap (Fig. 9A). Next, we selected these related genes as candidates for Gene Ontology (GO) and Kyoto Encyclopedia of Genes and Genomes (KEGG) enrichment analysis using Metascape [17]. GO analysis explains the functions of these genes through three aspects: cellular component (CC), molecular function (MF), and biological process (BP). The results of GO analysis show that genes regulated by METTL3 play an important role in cell division, differentiation, tissue development and maturation and immune regulation (Fig. 9B), which is basically consistent with the biological function of METTL3 [18–20]. KEGG analysis was used to explore the pathways involved in downstream genes regulated by METTL3. The most significant enrichment pathway is epithelial-mesenchymal transition (EMT), which is closely related to the occurrence and metastasis of lung cancer [21]. In addition, these genes are also widely involved in cellular responses to toxic substances, oxidative stress and other pathways, which may affect the tumor cell response to chemotherapy [22]. It is worth noting that METTL3 also regulates some pathways of the antitumor immune response through downstream genes, including peptidase activity and MHC protein complex binding. These pathways involve the processing and presentation of tumor antigens, which activate the immune system to produce antitumor effects (Fig. 9C) [23]. In conclusion, these analyses provide some clues for the contribution of METTL3 to the occurrence and prognosis of LUSC.

### Clinical sample verification

**Fig. 10:**
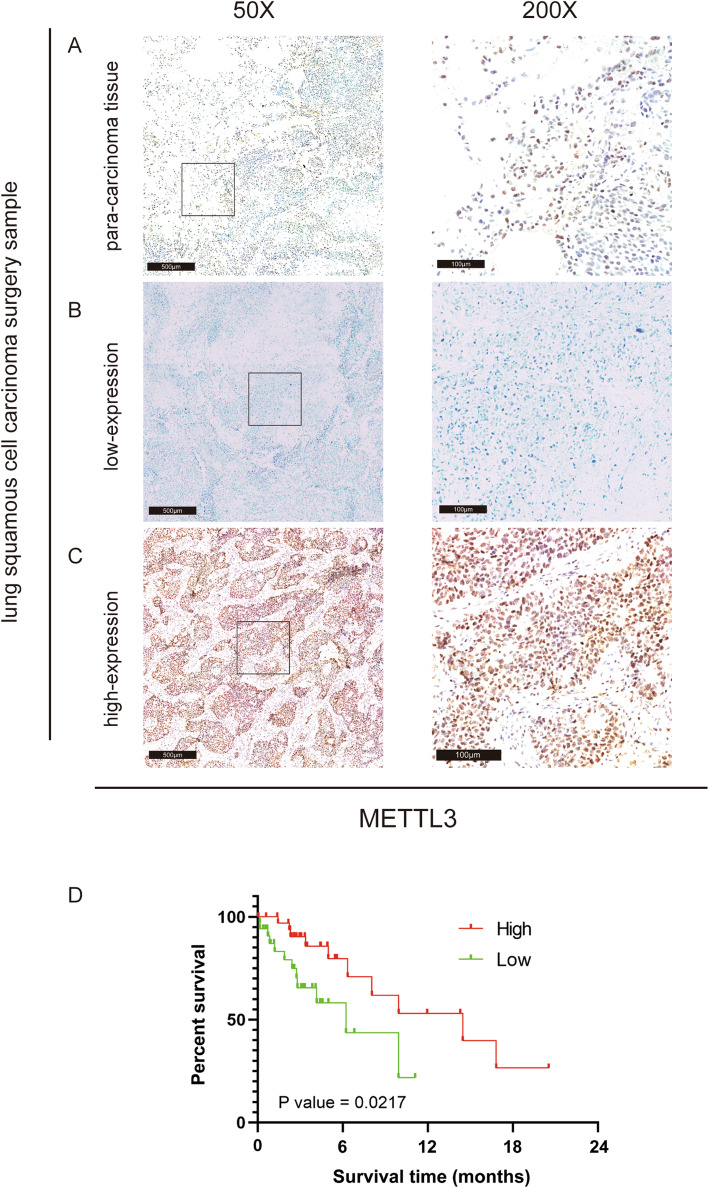
(A) Comparison of METTL3 expression in LUSC tissues and adjacent tissues. (B) Examples of patients with low METTL3 expression. (C) Examples of patients with high METTL3 expression. (D) Survival curves of LUSC patients with high and low METTL3 expression

To verify the above conclusions, we collected 70 surgical specimens of LUSC from the Affiliated Hospital of Qingdao University and performed immunohistochemical staining. As shown in Fig. 10, in most LUSC tissues, the immunoreactivity of METTL3 was positive. METTL3 expression in tumor tissues was significantly higher than that in adjacent tissues, which is consistent with the conclusion in Fig. 1, suggesting that METTL3 plays an important role in the development of LUSC *(*Fig. 10A*)*. Among these surgical specimens, 27 (38.6%) tissues were weakly stained (Fig. 10B) and the remaining 43 (61.4%) displayed strong staining (Fig. 10C). Finally, Kaplan-Meier curves with the log-rank test revealed that patients with high METTL3 expression exhibited a longer survival time than those with low METTL3 expression (Fig. 10D), suggesting that METTL3 can be used as a reliable prognostic biomarker for LUSC patients.

## Discussion

With the development of molecular biology techniques over the past few decades, researchers have gained increasing knowledge of lung cancer pathogenesis. Correspondingly, the rapid development of lung cancer treatment drugs will hopefully make lung cancer a chronic disease with a long survival time. Taking as an example lung adenocarcinoma accounting for the highest proportion of lung cancer, the rate of functional driver gene mutations including EGFR, KRAS and rearrangement of ALK or ROS1, is approximately 60% [24]. Compared with other lung cancer patients, the median survival time of lung adenocarcinoma with driver gene mutations as drug targets is prolonged by 2 to 4 years [25]. Studies have shown that LUSC has unique molecular biological characteristics. Its EGFR gene mutation rate is only 5.4%, and the incidence of ALK fusion genes is less than 2.5%, resulting in a lack of effective targeted therapy for LUSC [26, 27]. At present, the treatment of advanced LUSC is still platinum-based chemotherapy and the median survival time is approximately one year [5]. Therefore, we urgently need to conduct more in-depth research on the molecular biological mechanism of LUSC so that the corresponding targeted drugs can be developed as soon as possible to improve the survival time of LUSC patients.

In addition to classic gene mutations, the important role played by epigenetic changes such as RNA modifications in the occurrence and development of tumors has attracted increasing attention. The process of replacing the hydrogen atom attached to the sixth nitrogen atom of adenine on messenger RNA by methylation is called m^6^A, which is catalyzed by methyltransferases [28]. As the most common method of RNA regulation, m6A is involved in almost every process of RNA processing and metabolism from precursor mRNA processing and mRNA expression in the nucleus to mRNA translation and degradation in the cytoplasm [29–31]. As one of the components of methyltransferase, METTL3 directly affects the methylation level of various tumor-related mRNAs, thereby contributing to the occurrence, development and prognosis of tumors. Studies have shown that METTL3 is highly expressed in metastatic colorectal cancer and is related to poor prognosis [32]. Another research team found that in human lung adenocarcinoma cell lines, METTL3 can promote the translation of oncogenes, thereby promoting tumor cell survival, proliferation and invasion [11]. In this article, METTL3 expression in tumor tissues was significantly higher than that in normal tissues, and the results of KEGG analysis showed that METTL3 is highly correlated with the epithelial-mesenchymal transition pathway, which is similar to the abovementioned literature and suggests the cancer-promoting effects of METTL3. Interestingly, our results indicate that the high expression of METTL3 in LUSC patients is a signal of better prognosis, which seems to contrast existing views. Through analysis, we found that the expression of METTL3 is only related to the overall survival time of patients, and has nothing to do with the pathological stage, which suggests that METTL3 may affect the patient’s response to anti-tumor therapy and thus affect the prognosis. The results of KEGG analysis show that genes regulated by METTL3 are also widely involved in cellular responses to toxic substances, oxidative stress and antigen presentation pathways, which may affect the tumor cell response to chemotherapy, anti-vascular therapy and immune checkpoint inhibitors. Besides, there are precedents for similar things. For example, some researchers reported that in hepatocellular carcinoma, the expression of METTL14 is significantly downregulated, which reduces the overall m^6^A methylation level of RNA. Low-level methylation of mRNA causes the expression of the tumor suppressor molecule miR-126 in the mature body to decrease, thereby ultimately promoting the metastasis and invasion of hepatocellular carcinoma [33]. However, a year later, other researchers reached a different conclusion. The results showed that the proliferation and migration of hepatocellular carcinoma cells were inhibited after METTL14 was knocked down in these cells. Using the CRISPR-Cas9 system to increase the expression of METTL14 promoted the proliferation and migration of hepatocellular carcinoma cells [10]. The results of the two studies are quite different, which means that there are still more mechanisms needing to be explored to support these seemingly contradictory results in the field of RNA methylation. These controversies will push the studies of RNA methylation in a better direction.

In addition, current clinicians generally use the TNM stage as the basis for prognostic judgment. However, as a highly heterogeneous disease, the prognosis of lung cancer involves many factors. In many cases, patients with the same stage may also have a completely different prognosis. Traditional tumor staging focuses on tumor size and metastasis, while ignoring differences in gene and protein levels. Many studies explored the role of m^6^A methylation in the pathogenesis and prognosis of a variety of malignant tumors, but similar studies are rare in LUSC. In this article, we found the differential expression of m^6^A methyltransferase genes in tumor tissues through TCGA and GEO databases. More importantly, we distinguished two tumor subtypes based on METTL3 expression and constructed a prognostic model. The PCA and ROC curves verified the effectiveness of tumor subtypes and prognostic models, which were further confirmed in the test set. Finally, we believe that METTL3 may increase the antitumor activity of the immune system and sensitivity of tumor cells to chemotherapy or antivascular therapy by regulating antigen presentation, oxidative stress and cellular responses to toxic substances through KEGG analysis, thereby improving the prognosis of LUSC. We hope to provide some ideas for more detailed studies in the future to reveal the precise molecular mechanisms of METTL3 and the occurrence and prognosis of LUSC. At the same time, with the widespread application of next generation gene sequencing, we hope that METTL3 can provide patients and clinicians with more accurate tumor classification and prognosis judgments based on genetic testing. More accurate prognostic assessment can help physicians formulate individualized treatment for patients on the premise of reducing the waste of medical and financial resources. According to our classification, low-risk patients who may have a longer survival period deserve to be considered for more aggressive options such as immune checkpoint inhibitors.

It is worth noting that our experiment still has some unavoidable disadvantages. Due to the limited number of samples, we did not study the differential expression of METTL3 among various races and its impact on the prognosis of LUSC. There are obvious differences in gene expression profiles between different races. Second, the limited sample size will also affect our results. Finally, we did not clarify the specific mechanism by which METTL3 affects the prognosis of LUSC. In summary, we studied the relationship between the expression of METTL3 and the prognosis of LUCS. We hope that these findings provide clues for future scientific research and clinical practice.

## Conclusion

In this article, we systematically elaborated that the high expression of METTL3 in patients with lung squamous cell carcinoma can be used as an independent predictor of good prognosis. We infer that METTL3 can improve the responsiveness of patients to anti-tumor treatments including chemotherapy, anti-vascular therapy and immunotherapy through a variety of signaling pathways. Of course, its specific role and mechanism in lung squamous cell carcinoma need further study.

## Data Availability

All data generated during this study are included in this published article. The datasets used to generate the data in the current study are available from the TCGA and GEO database.

## Abbreviations

M6A: N^6^-methyladenine
LUSC: lung squamous cell carcinoma
TCGA: The Cancer Genome Atlas
GEO: Gene Expression Omnibus
PCA: principal component analysis
ROC: receiver operating characteristic
IHC: immunohistochemistry
AUC: Area Under Curve
NSCLC: nonsmall cell lung cancer
LUAD: lung adenocarcinoma
TKIs: tyrosine kinase inhibitors
mRNA: messenger RNA
METTL3: methyltransferase-like 3
METTL14: methyltransferase-like 14
WTAP: Wilms tumor I associated protein
GO: Gene Ontology
KEGG: Kyoto Encyclopedia of Genes and Genomes
EMT: epithelial-mesenchymal transition
